# Evidence of *Leishmania infantum* Infection in Rabbits (*Oryctolagus cuniculus*) in a Natural Area in Madrid, Spain

**DOI:** 10.1155/2014/318254

**Published:** 2014-03-03

**Authors:** Nerea García, Inmaculada Moreno, Julio Alvarez, María Luisa de la Cruz, Alejandro Navarro, Marta Pérez-Sancho, Teresa García-Seco, Antonio Rodríguez-Bertos, María Luisa Conty, Alfredo Toraño, Antonio Prieto, Lucas Domínguez, Mercedes Domínguez

**Affiliations:** ^1^Centro de Vigilancia Sanitaria Veterinaria (VISAVET), Universidad Complutense, 28040 Madrid, Spain; ^2^Departamento de Inmunología, Instituto de Salud Carlos III, Majadahonda, 28220 Madrid, Spain; ^3^Instituto Ramón y Cajal de Investigación Sanitaria (IRYCIS), Ctra. Colmenar Viejo, Km. 9.100, 28034 Madrid, Spain; ^4^Departamento de Medicina y Cirugía Animal, Facultad de Veterinaria, Universidad Complutense, 28040 Madrid, Spain; ^5^Madrid Salud, Ayuntamiento de Madrid, 28007 Madrid, Spain

## Abstract

Leishmaniasis is one of the most important neglected zoonosis and remains endemic in at least 88 developing countries in the world. In addition, anthropogenic environmental changes in urban areas are leading to its emergency world wide. Zoonotic leishmaniasis control might only be achieved by an integrated approach targeting both the human host and the animal reservoirs, which in certain sylvatic cycles are yet to be identified. Recently, hares have been pointed out as competent reservoirs of *Leishmania infantum* in Spain, but the role of other lagomorphs has not been clarified. Here, 69 rabbits (*Oryctolagus cuniculus*) from a natural area in Madrid in which a high density was present were analyzed using indirect (immunofluorescence antibody test, IFAT) and direct (PCR, culture) techniques. Fifty-seven (82.6%) of the animals were positive to at least one technique, with IFAT yielding the highest proportion of positive samples. *L. infantum* was isolated in 13% animals demonstrating the occurrence of infection in this setting. Our results suggest that rabbits could play a role of competent reservoir of *L. infantum* and demonstrate that the prevalence of infection is high in the analyzed area.

## 1. Introduction

Leishmaniasis is a world-wide vector-borne disease caused by protozoan parasites of the genus *Leishmania *that affect humans and other mammal reservoir hosts [1]. Nowadays leishmaniasis is considered an emerging disease in Central and Northern European countries [2, 3]. In the Mediterranean area, where leishmaniasis is endemic, *Leishmania infantum* is the agent responsible of the disease [4], and sandflies of the genus *Phlebotomus* (mainly* P. perniciosus*) are the main vectors [5–8] while the dog is considered the major domestic reservoir [9–11]. Zoonotic leishmaniasis control might only be achieved by an integrated approach targeting both the human host and the animal reservoirs, which in certain sylvatic cycles are yet to be identified. *Leishmania* infection in wild carnivores (mongooses, red foxes, genets, lynxes, wolves, etc.) and rodents has also been described in different countries [12–17] including Spain [18–20] but the role of these species in the epidemiological cycle of *L. infantum* is unclear. A *Leishmania*-infected mammal species that carries live parasites can be considered a suspected parasite reservoir, but in order to consider it as a proven reservoir its active role in the perpetuation of the disease must be fully demonstrated, including the use of xenodiagnostic studies [16].

However, as a result of recent investigations carried out after the leishmaniasis outbreak occurred in the south-west area of the province of Madrid (Spain) from 2009 to 2012, in which at least 446 human cases have been reported, the Iberian hare (*Lepus granatensis*) has been described as a new competent reservoir of the disease [21–23]. *L. infantum* infection in hares was evidenced using PCR analysis and indirect immunofluorescence antibody test (IFAT) [24], and its ability to transmit the parasite to sandflies (*P. perniciosus*) was demonstrated by xenodiagnostic studies [23]. This laboratory evidence, combined with epidemiological data, confirmed that Iberian hares (and not dogs) were contributing to the outbreak [22]. Another study performed in 94 hares trapped in different geographic regions of Spain found a prevalence of *L. infantum* infection of 43.6% based on DNA detection in spleen samples from principally two hare species (*L. granatensis* and *L. europaeus*) [25], highlighting the potential importance of these species in the epidemiology of leishmaniasis in Spain. In one of the studies assessing the importance of possible alternative reservoirs in the outbreak area of Madrid a seroprevalence of 46% was also reported in rabbits [24], therefore suggesting that hares may not be the only reservoir maintaining the infection in this setting.

In order to evaluate the possible risk posed by rabbits as a potential source of *L. infantum* infection, another study was carried out in a natural area of Madrid where a very high density of this species has been noted, using indirect (serology) and direct (PCR-detection, culture) diagnostic techniques.

## 2. Materials and Methods

A total of 69 European rabbits (*Oryctolagus cuniculus*) were captured for scientific purposes by convenience sampling by ferreting [26] during September 2013 in a public restricted natural area of approximately 80 hectares in Madrid (Central Spain) after receiving ethical clearance by the Health and Environment authorities of Madrid Council. Several favorable conditions for the vector reproductive cycle were present in the area of study, such as a high relative humidity, mean temperatures between 17 and 30°C and large accumulations of organic materials. In addition, the rabbit population in the area showed a substantial increase in the last years. Sample size was set to detect infection for a minimum expected prevalence of 5% with a level of confidence of 95% [27].

Rabbits were transported to the lab within the first five hours after their capture and were necropsied to observe macroscopic lesions compatible with subclinical infections. In addition information on the sex (determined by observing the external genitalia) and age (established based on the presence of cartilage conjunction in the ulna of the forelegs [28]) was available in 44 of the 69 animals (16 male and 28 female, 18 young, and 26 adult rabbits).

Serum, spleen, and skin (from the external ear) samples were collected from all animals for laboratory determinations. Sera were analyzed using IFAT; a portion of the spleen was used immediately for in vitro culture, while the rest of the spleen sample and the skin were stored at −20°C for subsequent PCR analyses.

### 2.1. IFAT Analysis

Rabbit serum titer against *L. infantum* was carried out as previously described [24]. Briefly, 24-well glass slides coated with 2 × 10*E*5  *L. infantum* (MCAN/ES/97/10,445) zymodeme MON-1 grown for 5 in vitro passages were used. Serum samples (10 *μ*L) were analyzed by serial doubling dilution (1/25 to 1/800) in PBS and incubated for 30 min at 37°C. Slides were washed three times (10 min each) in PBS, and 10 *μ*L of fluorescein-labeled goat anti-rabbit immunoglobulin (4050-02; Southern Biotech, AL, USA) diluted in PBS supplemented with Evans blue (diluted 1/10^4^) were added to wells and incubated (37°C, 30 min). After incubation, slides were washed three times in PBS, mounted, and examined in a fluorescence microscope (Zeiss Axioskop 40; 40x magnification). To detect anti-*Leishmania* antibodies, a threshold value was established at 1/25 dilution (at this dilution, background antitrypanosomatid reactivity due to natural antibodies was negligible) using sera from *Leishmania*-seronegative naïve NZW rabbits as described before [24]. As a species-specific target antigen, we used *L. infantum* promastigotes derived from various culture passages. For genus-specific antigen controls, low-passage *L. amazonensis* promastigotes were used in parallel.

### 2.2. PCR Analysis

#### 2.2.1. DNA Extraction

A portion of approximately 10 mg of spleen and 25 mg of skin was placed in 300 *μ*L of NET-10 buffer. DNA extraction was performed using the QIAamp Blood and Tissue kit (QIAGEN, Hilden, Germany) according to the procedure recommended by the manufacturer. DNA was resuspended in 150 *μ*L of elution buffer and frozen at −80°C until use.

#### 2.2.2. PCR

A specific *Leishmania* nested PCR reaction aimed at the SSU-rRNA region [29] was performed in all the skin and spleen samples. Negative (sterile water) and positive controls [DNA obtained from *L. infantum* (MCAN/ES/97/10,445) zymodeme MON-1 promastigotes] were used in each assay. The reactions were carried out in I-Cycler thermocycler (Bio-Rad) equipment. PCR products were visualized into a 3% agarose (Ultrapure Agarose, Invitrogen) gel using DNA SYBR Safe gel stain (Invitrogen) and 1 ul of loading marker (Promega, WIS, USA; 100 kb ladder) was used. Positive samples yielded a PCR product of 358 pb.

### 2.3. Limiting Dilution Analysis

Spleens were excised aseptically, weighted, and a piece of ~10 mg was taken to determine parasite DNA by PCR analysis. The remaining spleen tissue was homogenized with a tissue grinder in Schneider's drosophila medium supplemented with 10% heat inactivated fetal calf serum (Hy Clone, Thermo Fisher Scientific, Waltham, MA, USA) penicillin (100 U/mL), streptomycin (50 mg/mL), (Lonza, Basel, Switzerland) 20 mM HEPES (Sigma-Aldrich, St. Louis, MO, USA), and 1% sterile urine, and the homogenate was adjusted to 10 mg of tissue/mL of medium. Eight aliquots of 0.15 mL were seeded in the first column of a 96-well culture plate, and then a series of six 1/4 serial dilutions (from 1 to 1/1024) were made. The plate was incubated at 27°C for 15 days. Plates were visualized for promastigote growth with an inverted microscope at 200x magnification. Parasite burden was calculated from the reciprocal of the highest dilution at which promastigotes were observed [30].

### 2.4. Statistical Analyses

Proportions of positive samples to each technique and individual characteristics, when available (age, sex) were compared using chi-square and Fisher exact tests. Agreement between tests was measured using the Kappa statistic. All calculations were carried out using the SPSS software V.20 (IBM Inc., Chicago, Il, USA).

## 3. Results and Discussion

Different diagnosis techniques were used to confirm *Leishmania* infection as none of them could be considered as a “gold standard” tool [31]. Overall 52 (75.4%) out of the 69 rabbits were considered positive in the IFAT (Table 1), with titers ranging from 1/25 to 1/800 (81.2% of the seropositive samples had titers of 1/100 or higher). Reactivity to conserved *Leishmania* spp. epitopes was, on average, two titer steps lower than to that *L. infantum*, showing a preferential antibody response to epitopes of the infecting species. Our results are in contrast with a previous study performed in wild rabbits in southeastern Spain, in which a seroprevalence of 0% was estimated analyzing 36 plasma samples with a modified commercial ELISA, although one animal was positive using direct PCR on tissues [32]. The seroprevalence in rabbits found in this population was also higher than that recently described in the outbreak area (45.7%) with the same serological test used here [24]. The proportion of seropositive animals was similar in adult (76.9%) and young rabbits, as well as in males (81.2%) and females (75.0%) and therefore no statistically significant association between these variables and the test result was observed (*P* > 0.4), in agreement with previous results in wild carnivores in Spain [20].

No macroscopic lesions in liver or spleen were observed in all animals after postmortem examination, suggesting a limited clinical impact of the infection in the hosts as described before for wild hosts other than domestic host [33].

PCR analysis of skin samples detected 12 (17.4%) positive samples, while *Leishmania* DNA was only found in two (2.9%) spleen samples (inconclusive results were found in another animal) (Table 1). One animal was positive simultaneously in both spleen and tissue samples, while another spleen-positive rabbit had a negative PCR-result in the skin. Seven (58.3%) and 45 (78.9%) of the 12 and 57 skin-PCR positive and negative animals, respectively, were also positive in the IFAT, suggesting a lack of association between the results in these tests (Fisher's exact test, *P* = 0.15) (Table 2). Again, no significant association was found between the results of the molecular analysis and the age/sex of the animals.

*L. infantum* was isolated from the spleen of 9 (13%) of the 69 rabbits (Table 1). The parasitic load ranged from 506 to more than 10,217 parasites per organ (spleen), a load approximately 10 times lower than that observed in experimental infections in mice [30]. No significant association between the culture results and those from the IFAT/PCR was observed (Fisher's exact test, *P* = 1.0).

Agreement between the three techniques performed here (IFAT, direct PCR, and isolation of the parasite) on different samples (serum, spleen, and skin) was very low (Kappa < 0.2 in all cases) (Table 2). The highest proportion of positive samples was observed using IFAT. False positive results in serological tests such as IFAT may occur due to the imperfect specificity of the tests and the presence of cross-reacting antigens, although the IFAT is considered a highly specific test for diagnosis of *Leishmania* infection [34, 35]. Comparison of the results obtained using antigens from *L. infantum* and *L. amazonensis* confirms the specificity of the reactions detected in animals considered as positive here. In addition, the isolation of *L. infantum* in 13% of the analyzed spleen samples, the first isolation of this parasite in the rabbit to the authors' knowledge, confirms the presence of a true infection of the rabbits in the area under study. Differences in the results of PCRs performed on spleen and skin samples have been reported before and may be related with the uneven distribution of *Leishmania* across tissues and the analysis of individuals in different stages of infection [36]. The higher proportion of culture positive spleen samples compared to PCR (performed on spleen) could be explained at least in part by the higher amount of tissue used for the former technique coupled with the low parasitic load found (other techniques as spleen imprints are considered negative if the parasite burden is below 10^5^) [30].

If the four diagnostic tests are interpreted in parallel (i.e., considering an animal as infected if a positive result was found in any test) 57 (82.6%) out of the 69 analyzed individuals would be considered infected. This percentage is higher than those described using different techniques for other potential *L. infantum* reservoirs such as fox (14.1%) and wolf (20.5%) in peninsular Spain [20], and genet (10%), feral cat (26%), and pine marten (39%) in endemic areas of Mallorca Island [19]. However, differences may be also attributed at least in part to the use of different diagnostic techniques (IFAT, direct PCR on spleen and skin samples and culture applied in parallel compared with direct PCR on spleen or blood samples and Western blotting-based serology). A number of epidemiological studies have determined anti-*L. infantum* seroprevalence in the cat using in-house developed IFAT techniques, although sensitivity and specificity parameters were not determined [37–40]. There is thus a clear need to evaluate and optimize IFAT as an analytical method for epidemiological use in noncanine species.

Our results are comparable to those determined in hares in a recent leishmaniosis outbreak in Madrid (considered as a local important reservoir of disease) by direct PCR (43.5%) and IFAT (74.1%) [24]. This could be due to the confluence of certain epidemiological factors favoring leishmaniosis transmission such as an increased density in the host and vector populations coupled by the lack of predators or other competent hosts for the parasite, similar to what was described in the outbreak area in Madrid [41].

## 4. Conclusions

Our results demonstrate that *L. infantum* infection is present in the rabbit population in Madrid as evidenced by the detection of specific antibodies against the parasite and *Leishmania* DNA and, especially, by the first isolation of this protozoon from rabbit. The lack of macroscopical lesions observed in spleen and liver of positive animals suggest that infection may remain asymptomatic and, given the high proportion of positive individuals found using all techniques, highlights the potential importance of this host species as an alternative reservoir of infection as already described for hares in Spain. Further studies are needed to determine the role of rabbits in the epidemiology of leishmaniasis.

## Figures and Tables

**Table 1:** Results of indirect immunofluorescence antibody test (IFAT), direct PCR for detection of parasite DNA and parasite isolation performed for detection of *Leishmania infantum* infection on 69 rabbits (*Oryctolagus cuniculus*) collected on a natural area in Madrid.

Test	Sample	Positive samples	% of positive samples
			(95% Confidence intervalI)
IFAT	Sera	52	75.4 (64.0–84.0)
Direct PCR	Spleen	2	2.9 (0.8–10.0)
	Skin	12	17.4 (10.2–2.8)
Isolation	Spleen	9	13.0 (7.0–23.0)

**Table 2:** Agreement between the results obtained in the indirect immunofluorescence antibody test (IFAT) and PCR on skin samples/parasite isolation from spleen samples performed in 69 rabbits (*Oryctolagus cuniculus*) from a natural area in Madrid.

IFAT	PCR on skin sample		Isolation		Total
	Positive	Negative	Positive	Negative	
Positive	7	45	7	45	**52**
Negative	5	12	2	15	**17**
Total	**12**	**57**	**9**	**60**	**69**
